# Intraperitoneal Instillation of Local Anesthetic (IPILA) in Bariatric Surgery and the Effect on Post-operative Pain Scores: a Randomized Control Trial

**DOI:** 10.1007/s11695-022-06086-w

**Published:** 2022-05-04

**Authors:** Ramandeep Kaur, Alexa Seal, Igor Lemech, Oliver M. Fisher, Nicholas Williams

**Affiliations:** 1 School of Medicine Sydney, Rural Clinical School (Wagga Wagga), The University of Notre Dame Australia, 40 Hardy Avenue, PO Box 5050, Wagga Wagga, NSW 2650 Australia; 2 Anesthetic Department, Calvary Hospital Riverina, 26-36 Hardy Avenue, Wagga Wagga, NSW 2650 Australia; 3 Upper Gastrointestinal Surgery, Department of Surgery, St George Hospital, Gray St, Kogarah, NSW 2217 Australia

**Keywords:** Bariatric surgery, Intraperitoneal instillation, Ropivacaine, Post-operative pain

## Abstract

**Background:**

Effective analgesia after bariatric procedures is vital as it can reduce post-operative opioid use. This leads to less nausea which may be associated with shorter post-operative length of stay (LOS). Understanding analgesic requirements in patients with obesity is important due to the varied physiology and increased number of comorbidities.

**Objectives:**

The aim of this study was to evaluate the efficacy of intraperitoneal instillation of local anesthetic (IPILA) to reduce opioid requirements in patients undergoing laparoscopic bariatric surgery.

**Methods:**

A double-blinded randomized control trial was conducted to compare intraperitoneal instillation of ropivacaine to normal saline in 104 patients undergoing bariatric surgery. The primary endpoint was pain in recovery with secondary endpoints at 1, 2, 4, 6, 24, and 48 h post-operatively. Further endpoints were post-operative analgesic use and LOS. Safety endpoints included unexpected reoperation or readmission, complications, and mortality.

**Results:**

There were 54 patients in the placebo arm and 50 in the IPILA. Pain scores were significantly lower in the IPILA group both at rest (*p* = 0.04) and on movement (*p* = 0.02) in recovery with no difference seen at subsequent time points. Equally, IPILA was independently associated with reducing severe post-operative pain at rest and movement (adjusted odds ratio [aOR] 0.28, 95% CI 0.11–0.69, *p* = 0.007 and aOR 0.25, 95% CI 0.09–0.62, *p* = 0.004, respectively). There was no significant difference in LOS, opioid use, antiemetic use, morbidity, or mortality between the intervention and placebo groups.

**Conclusion:**

The administration of ropivacaine intraperitoneally during laparoscopic bariatric surgery reduces post-operative pain in the recovery room but does not reduce opioid use nor LOS.

**Supplementary Information:**

The online version contains supplementary material available at 10.1007/s11695-022-06086-w.

## Introduction

Laparoscopy has greatly reduced the pain and recovery of surgery; however, optimizing post-operative pain is an ongoing goal of management. Despite improved techniques, some patients continue to experience significant post-operative pain requiring strong opioids [1]. Post-operative pain after laparoscopic bariatric procedures arises from port site insertion, visceral pain from stretching of the peritoneum during gas insufflation and the procedure itself, and diaphragmatic irritation resulting in shoulder tip pain. Post-operative pain prolongs patient hospitalization leading to additional costs [2–4]. Furthermore, post-operative opioid use can lead to related adverse effects such as nausea and vomiting which may decrease oral intake thus leading to delayed discharge and increasing length of stay (LOS). Hence, improved post-operative pain control may in turn reduce opioid use thus potentially decreasing LOS.

The safety and efficacy of local anesthetic in perioperative care is well recognized. The primary advantages of local anesthetic agents are that they act directly on the tissue to which they are administered and they lack the systemic effects of opioids, such as nausea, sedation, and opioid-induced ventilatory impairment [5–7]. Analgesia requirements in patients with obesity can vary due to differing physiology and comorbidities; therefore, understanding analgesia administration in bariatric procedures and utilizing a multimodal approach is particularly important. There have been studies conducted on the effectiveness of intraperitoneal local anesthetic in laparoscopic procedures including bariatric surgery. However, results from such studies are inconclusive; some demonstrated a reduction in post-operative pain [8, 10, 12–15, 18, 19] yet others found no difference [9, 11, 16, 17]. Previous studies conducted on intraperitoneal local anesthetic in bariatric patients have administered a standardized volume of local anesthetic to every patient. However, how varying patient weights may influence local anesthetic efficacy remains unclear. The aim of this study was to evaluate the efficacy of weight-standardized intraperitoneal instillation of local anesthetic (IPILA) in patients undergoing laparoscopic bariatric surgery and determine whether it can affect post-operative pain outcomes.

## Methods

This was a randomized control trial which was registered with ANZCTR (ACTRN 12,618,000,389,202) and was approved by both Calvary Hospital Wagga Wagga administration and the local University of Notre Dame Australia review board (Reference number: 018114S). The full trial protocol can be accessed on www.anzctr.org.au. Written informed consent was obtained from all participants. Study sample size has been calculated using a standardized effect size of 0.6 using the Cohen’s *d* test and evidence from previous studies [13–16, 20]. The constant used was 0.79 based on *p* < 0.05 and a power of 80%. A sample size of 100 patients was calculated with 50 in each arm. Twenty percent oversampling was performed to account for missing pain score and follow-up data.

### Participants

Adult patients undergoing laparoscopic bariatric surgery [sleeve gastrectomy (SG), one anastomosis gastric bypass (OAGB), Roux-en-Y gastric bypass (RYGB), single anastomosis duodenal-ileal bypass (SADI), and revision surgery] were identified and recruited between November 2018 and November 2020. Patients were excluded if they had an allergy to local anesthetic, severe cardiovascular disease (congestive heart failure or ischemic heart disease), chronic renal disease (creatinine clearance < 60 mL/h), Child–Pugh score B/C, or if they declined to participate.

### Surgery

All patients underwent their procedure with the same surgeon and anesthetist at a single institution. Laparoscopic procedures were typically carried out using a 12-mm optical entry camera port, two 12–15-mm operating ports, a 5-mm Nathanson liver retractor port, and a 5-mm assistant port. Carbon dioxide insufflation was set to a pressure of 14 mmHg. At the end of each case, a mixing cannula was used to spray a solution of either normal saline or 0.2% ropivacaine onto the diaphragm prior to the conclusion of the procedure. The amount of solution to be instilled was 0.5 mL/kg calculated based on the patient weight on the morning of the procedure to ensure safety and efficacy of the local anesthetic.

### Anesthetic Protocol

Patients were given a relaxant general anesthetic. Standardized monitoring, including 3-lead electrocardiogram, non-invasive blood pressure (NIBP), oxygen saturation, and neuromuscular monitoring, was attached. Invasive arterial monitoring was used only in the case of poorly fitting or grossly inaccurate NIBP. Patients were positioned in reverse Trendelenburg. Induction proceeded with fentanyl, ketamine, propofol, and rocuronium. An endotracheal tube was introduced once deep muscle relaxation was confirmed. The stomach was decompressed with a temporary orogastric tube. Anesthesia was maintained with oxygen/air/sevoflurane with FiO_2_ of 40%. During the case, the patient was converted over to a combination intravenous propofol/desflurane maintenance with the view to facilitating early respiration and quicker extubation. Each patient received dexamethasone (8 mg) at the beginning of the case, unless they were diabetic on oral hypoglycaemic agents or insulin. Parecoxib (40 mg), droperidol (0.625 mg), and ondansetron (4 mg) were administered at the conclusion of the case. Paracetamol (2 g) was given on arrival in the post-anesthesia care unit (PACU) and there was as necessary (PRN) use of antiemetics and oxycodone, or fentanyl if there were allergies as required. All patients received standardized therapy on the ward which was recorded and consisted of PRN ondansetron 4 mg QID and tapentadol 50 mg PRN q3h (maximum dose 300 mg) and tramadol 50–100 mg IV/PO PRN QID. Patients were monitored in a ward-based setting with continuous saturation monitoring. A summary of anesthetic data is provided in Supplementary Table 1.

### Randomization and Blinding

Participants were randomized using block permutation method in a 1:1 ratio between the control and intervention arms. All individuals involved in the trial (patients, surgeons, nurses, and anesthetists) were blinded to treatment allocation. A member of the research team who was not directly involved in any aspect of the intervention and procedure was unblinded and responsible for randomization, preparation of solutions, and collection of survey forms. The allocation sequence was implemented using sequentially numbered solution bags.

### Outcome Measures

Pain scores were recorded by nursing staff using a visual analogue scale (VAS) at rest and on movement in the post-operative acute care unit (PACU) and at 1, 2, 4, 6, 24, and 48 h. Pain scores at 48 h were not recorded if the patient had been discharged. The primary endpoint was post-operative pain using VAS score in recovery both at rest and upon movement. Equally, the effect of IPILA on extremes of pain (VAS > 7) in PACU was assessed. Secondary endpoints included assessment of pain scores at subsequent time points until discharge. Other secondary efficacy endpoints were post-operative analgesia and antiemetic use and LOS. Safety endpoints were unexpected reoperation or readmission, complications, and mortality.

### Statistical Analysis

Continuous data for primary and secondary endpoints are summarized as medians with interquartile range or means with 95% CIs depending on their baseline normal distribution. The primary endpoint of pain scores between treatment and control groups was analyzed using Wilcoxon-rank sum test and/or chi-squared test as required. Secondary endpoints were analyzed using Student’s independent *t*-test or non-parametric alternative (Mann–Whitney test) and Pearson chi-square test as appropriate. Fisher’s exact test was used for safety endpoints such as reoperation or readmission, complications, and mortality due to the low number of events recorded. Uni- and multivariable linear and logistic regression analyses were performed to assess for the independent effect of IPILA on recorded post-operative pain perception. Results included unadjusted (univariable) and adjusted (multivariable) odds ratio with 95% CI estimates. Potential confounders such as surgery type, hiatus hernia repair, age, BMI, and chronic pain were controlled for in regression analyses. Data analysis was conducted using SPSS version 20 (Chicago, IL, USA) and R Statistical Programming.

## Results

**Fig. 1:**
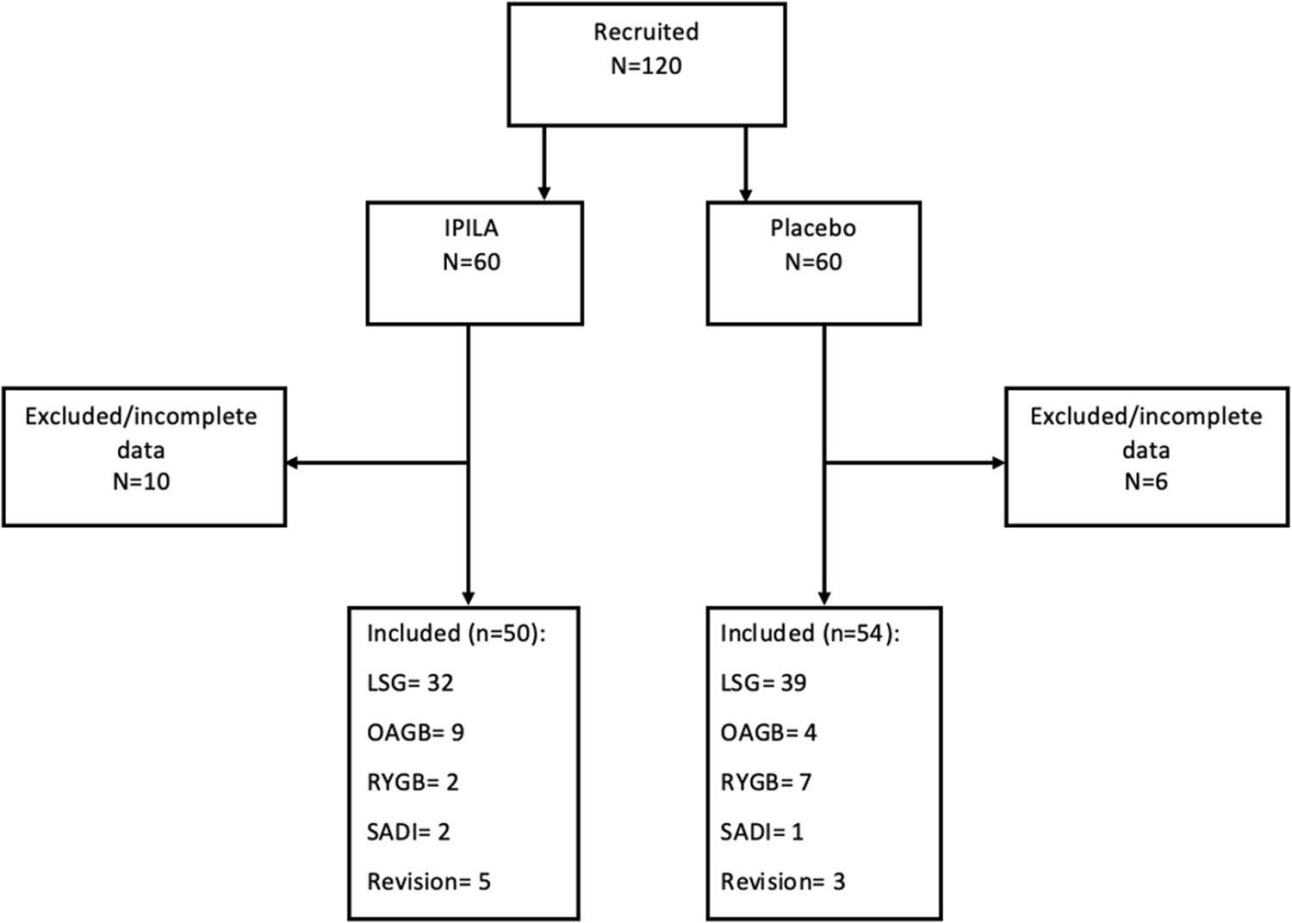
Recruitment and allocation flow diagram. IPILA, intraperitoneal instillation of local anesthetic

Of the 120 patients who were randomized, 104 were included in the final analysis, 54 in the placebo arm and 50 in the treatment arm (Fig. 1). Baseline demographics did not differ significantly between the two groups (Table 1). Majority of patients underwent SG in both groups. Five patients in each of the placebo and IPILA groups had been prescribed regular analgesic medications for preoperative chronic pain management. The three revision procedures in the placebo group were all RYGB. In the IPILA group, revision procedures were three RYGB, an OAGB, and a SADI.

**Table 1:** Baseline characteristics of patients in the placebo and treatment (IPILA) groups

	Total (*N* = 104)	IPILA (*N* = 50)	Placebo (*N* = 54)	*p*-value
Patient gender [*n* (%)]				0.782
F	82 (78.8)	40 (80.0)	42 (77.8)	
M	22 (21.2)	10 (20.0)	12 (22.2)	
Patient age (years), median (IQR)	41.0 (29.8–50.0)	44.0 (30.3–49.8)	34.0 (28.0–49.0)	0.099
Patient weight (kg), median (IQR)	115.8 (101.6–131.5)	117.9 (106.0–131.6)	111.6 (101.4–130.7)	0.540
Body mass index (kg/m^2^), median (IQR)	40.9 (36.8–46.1)	42.5 (36.8–46.5)	39.3 (36.7–44.9)	0.388
ASA, median (IQR)	3.0 (3.0–3.0)	3.0 (3.0–3.0)	3.0 (3.0–3.0)	0.889
Ethnicity [*n* (%)]				0.229
Aboriginal/Torres Strait Islander	10 (9.6)	3 (6.0)	7 (13.0)	
Caucasian	94 (90.4)	47 (94.0)	47 (87.0)	
Non-smoker [*n* (%)]	104 (100.0)	50 (100.0)	54 (100.0)	0.695
Preoperative chronic pain [*n* (%)]	11 (10.6)	6 (12.0)	5 (9.3)	0.650
Preoperative pain medication use [*n* (%)]	12 (11.5)	6 (12.0)	6 (11.1)	0.887
Preoperative opioid use [*n* (%)]	6 (5.8)	4 (8.0)	2 (3.7)	0.348
Preoperative non-opiod use [*n* (%)]	9 (8.7)	5 (10.0)	4 (7.4)	0.638
Surgery type [*n* (%)]				0.204
LSG	73 (70.2)	33 (66.0)	40 (74.1)	
OAGB	13 (12.5)	9 (18.0)	4 (7.4)	
RYGB	14 (13.5)	5 (10.0)	9 (16.7)	
SADI	4 (3.8)	3 (6.0)	1 (1.9)	
Revision surgery [*n* (%)]	8 (7.7)	5 (10.0)	3 (5.6)	0.395
Concomitant hiatus hernia repair [*n* (%)]	26 (25.2)	17 (34.7)	9 (16.7)	0.035
Length of stay (days), median (IQR)	1.0 (1.0–2.0)	1.0 (1.0–2.0)	1.0 (1.0–2.0)	0.631

*IPILA*, intraperitoneal instillation of local anesthetic; *LSG*, laparoscopic sleeve gastrectomy; *OAGB*, one anastomosis gastric bypass; *RYGB*, Roux-en-Y gastric bypass; *SADI*, single anastomosis duodenal-ileal bypass

### Primary Endpoint Analysis

There was a significant difference in pain scores between the two groups in PACU with the IPILA group having lower pain scores both at rest (median VAS 5.0 [2.25–6.0] IPILA vs. median VAS 6.0 [5.0–8.0] placebo, *p* = 0.04) and on movement (median VAS 5.0 [IQR 3.0–7.0] IPILA vs. median VAS 7.0 [IQR 5.0–8.0], *p* = 0.019). Patients receiving IPILA had less severe pain episodes compared to placebo at rest (VAS ≥ 7 22% in the IPILA group vs. 48.1% in the placebo, *p* = 0.005) and on movement (VAS ≥ 7 28% in the IPILA group vs. 59% in the placebo, *p* = 0.002, Table 1). Uni- and multivariable linear and logistic regression analyses were performed to control for potential confounding. Whilst no significant independent effect of IPILA was found on overall mean VAS scores at resting or on movement (Supplementary Tables 2 and 3), an independent effect on reduction of high pain scores (VAS ≥ 7) could be documented at both rest and on movement (adjusted odds ratio [aOR] 0.28, 95% CI 0.11–0.69, *p* = 0.007 and aOR 0.25, 95% CI 0.09–0.62, *p* = 0.004, respectively, Tables 2, 3, and 4).

**Table 2:** Median visual analogue scale (VAS) for IPILA and placebo groups post-bariatric surgery and proportion of patients with extreme pain (VAS ≥ 7)

Time point	Action	Median VAS (95% confidence interval)			*p*-value
		Total	IPILA	Placebo	
PACU	Resting	5.0 (3.0–7.0)	5.0 (3.0–6.0)	6.0 (5.0–8.0)	0.04
	Movement	6.0 (3.0–8.0)	5.0 (3.0–7.0)	7.0 (5.0–8.0)	0.02
PACU *n *(%)	Resting VAS < 7	67 (64.4%)	39 (78.0%)	28 (51.9%)	0.005
	Resting VAS ≥ 7	37 (35.6%)	11 (22.0%)	26 (48.1%)	
PACU *n *(%)	Movement VAS < 7	58 (56.3%)	36 (72.0%)	22 (41.5%)	0.002
	Movement VAS ≥ 7	45 (43.7%)	14 (28.0%)	31 (58.5%)	
1 h	Resting	4.0 (2.0–6.0)	3.0 (2.0–5.8	4.0 (2.0–6.0)	0.98
	Movement	3.0 (2.0–5.0)	3.0 (2.0–4.0)	3.0 (2.0–5.0)	0.74
2 h	Resting	3.0 (1.3–5.0)	3.0 (2.0–5.0)	4.0 (1.0–5.0)	0.76
	Movement	4.0 (2.0–5.0)	4.0 (2.5–5.0)	4.0 (2.0–6.0)	0.71
4 h	Resting	2.0 (1.0–4.0)	2.0 (1.0–4.0)	2.5 (1.0–4.0)	0.99
	Movement	3.0 (2.0–6.0)	3.0 (2.0–5.0)	3.0 (2.0–6.0)	0.78
6 h	Resting	2.0 (1.0–4.0)	2.0 (1.0–4.0)	2.0 (2.0–3.5)	0.93
	Movement	4.0 (2.0–5.0)	3.0 (2.0–5.0)	4.0 (2.5–5.0)	0.31
24 h	Resting	2.0 (1.0–4.0)	2.0 (0.8–3.0)	2.0 (1.0–4.3)	0.23
	Movement	3.0 (2.0–5.0)	3.0 (2.0–4.0)	4.0 (2.0–5.0)	0.52
48 h	Resting	2.0 (1.0–2.0)	2.0 (0.0–2.0)	2.0 (1.0–2.0)	0.61
	Movement	3.5 (2.0–5.0)	3.0 (2.0–5.0)	4.0 (2.0–5.0)	0.73

PACU, post-operative acute care unit; *IPILA*, intraperitoneal instillation of local anesthetic; *VAS*, visual analogue scale

**Table 3:** Uni- and multivariable regression analyses of factors contributing to reduced extremes of pain as measured in post-operative acute care unit (PACU) at rest

				Univariable		Multivariable	
		**VAS < 7**	VAS ≥ 7	aOR (95% CI)	*p-*value	aOR (95% CI)	*p*-value
IPILA *n* (%)	No	28 (41.8)	26 (70.3)	Ref	-	Ref	-
	Yes	39 (58.2)	11 (29.7)	0.30 (0.13–0.70)	0.006	0.28 (0.11–0.69)	0.007
Surgery type *n* (%)	LSG	47 (70.1)	26 (70.3)	Ref	-	Ref	-
	OAGB	8 (11.9)	5 (13.5)	1.13 (0.31–3.75)	0.844	1.49 (0.37–5.62)	0.561
	RYGB	9 (13.4)	5 (13.5)	1.00 (0.28–3.23)	0.994	0.94 (0.23–3.55)	0.922
	SADI	3 (4.5)	1 (2.7)	0.60 (0.03–4.98)	0.668	0.66 (0.03–6.64)	0.747
Hiatus hernia repair *n *(%)	No	48 (72.7)	29 (78.4)	Ref	-	Ref	-
	Yes	18 (27.3)	8 (21.6)	0.74 (0.27–1.86)	0.527	1.11 (0.36–3.33)	0.858
Age, mean (SD)		40.6 (12.3)	38.8 (12.0)	0.99 (0.95–1.02)	0.463	0.99 (0.95–1.04)	0.797
BMI, mean (SD)		41.4 (6.5)	42.2 (6.6)	1.02 (0.96–1.08)	0.555	1.04 (0.97–1.12)	0.322
Chronic pain *n* (%)	No	60 (89.6)	33 (89.2)	Ref	-	Ref	-
	Yes	7 (10.4)	4 (10.8)	1.04 (0.26–3.70)	0.954	1.16 (0.24–5.29)	0.845

*IPILA*, intraperitoneal instillation of local anesthetic; *VAS*, visual analogue scale; *OR*, odds ratio; *CI*, confidence interval; *SD*, standard deviation; *BMI*, body mass index; *LSG*, laparoscopic sleeve gastrectomy; *OAGB*, one anastomosis gastric bypass; *RYGB*, Roux-en-Y gastric bypass; *SADI*, single anastomosis duodenal-ileal bypass

**Table 4:** Uni- and multivariable regression analyses of factors contributing to reduced extremes of pain as measured in post-operative acute care unit (PACU) on movement

				Univariable		Multivariable	
		VAS < 7	VAS ≥ 7	aOR (95% CI)	*p*-value	aOR (95% CI)	*p*-value
IPILA *n* (%)	No	22 (37.9)	31 (68.9)	Ref	-	Ref	-
	Yes	36 (62.1)	14 (31.1)	0.28 (0.12–0.62)	0.002	0.25 (0.09–0.62)	0.004
Surgery type *n* (%)	LSG	40 (69.0)	32 (71.1)	Ref	-	Ref	-
	OAGB	6 (10.3)	7 (15.6)	1.46 (0.44–4.95)	0.533	2.58 (0.69–10.20)	0.162
	RYGB	9 (15.5)	5 (11.1)	0.69 (0.20–2.22)	0.547	0.93 (0.22–3.83)	0.925
	SADI	3 (5.2)	1 (2.2)	0.42 (0.02–3.43)	0.458	0.32 (0.01–3.58)	0.389
Hiatus hernia repair *n *(%)	No	40 (70.2)	36 (80.0)	Ref	-	Ref	-
	Yes	17 (29.8)	9 (20.0)	0.59 (0.23–1.46)	0.261	1.35 (0.44–4.22)	0.603
Age, mean (SD)	(SD)	42.7 (12.1)	36.7 (11.5)	0.96 (0.92–0.99)	0.015	0.96 (0.92–1.00)	0.080
BMI, mean (SD)	(SD)	41.0 (6.5)	42.9 (6.4)	1.05 (0.98–1.12)	0.148	1.07 (0.99–1.17)	0.078
Chronic pain *n* (%)	No	51 (87.9)	41 (91.1)	Ref	-	Ref	-
	Yes	7 (12.1)	4 (8.9)	0.71 (0.18–2.52)	0.606	1.00 (0.19–4.96)	0.996

*IPILA*, intraperitoneal instillation of local anesthetic; *VAS*, visual analogue scale; *OR*, odds ratio; *CI*, confidence interval; *SD*, standard deviation; *BMI*, body mass index; *LSG*, laparoscopic sleeve gastrectomy; *OAGB*, one anastomosis gastric bypass; *RYGB*, Roux-en-Y gastric bypass; *SADI*, single anastomosis duodenal-ileal bypass

### Secondary Endpoint Analysis

No significant difference was seen between the IPILA and placebo groups at other post-operative time points, in post-operative analgesia intake, antiemetics use, nor mean LOS. The mean frequency of requests for opioid analgesia was 1.8 (95% CI 1.5–2.1) per hospitalization in the placebo group and 1.9 (95% CI 1.56–2.29) in the IPILA group. Mean use of antiemetics was 1.5 times per admission in the placebo group (95% CI 0.1–0.7) and 1.9 times (95% CI 0.2–0.6) in the IPILA group (*p* = 0.270). The median LOS was 1.0 days in both the placebo (IQR 1.0–2.0) and IPILA groups (IQR 1.0–2.0, *p* = 0.63). In addition, there were no significant differences between the groups with respect to the safety endpoints. There were no unexpected reoperations, no unplanned ICU admission, and no mortality. The only complication was a pulmonary embolus (PE) in one patient in the IPILA arm. There were five unplanned readmissions in the IPILA group and four in the placebo group, all for patients who required IV rehydration.

## Discussion

In this study, patients who received IPILA had significantly lower pain scores in PACU. Equally, IPILA was independently associated with a reduction in the likelihood of patients experiencing extreme pain scores. Although not significant, pain scores were also lower at all other time points in the IPILA group up to 4 h post-surgery. This is consistent with the half-life of ropivacaine being 4.2 h [20] by which stage the patients have progressed from recovery to ward-based care. It is also important to consider possible confounders and their potential impact on post-operative outcomes. Uni- and multivariable linear and logistic regression analyses were conducted in order to control for multiple variables including surgery type, hiatus hernia repair, age, BMI, and chronic pain when determining the effect of the IPILA intervention. These analyses showed IPILA was the only variable which had significantly lower adjusted odds ratios of severe pain episodes in PACU at rest and on movement. We hypothesized that a reduction in pain immediately post-operatively allows for reduced administration of opioids and hence nausea, facilitating quicker recovery and shorter LOS. However, this was not evident in the results with total opioid use, antiemetic use, and LOS being equivalent between the two groups. This is perhaps explained by an already short mean LOS of only 1 day.

Studies have been conducted on several bariatric procedures such as SG, RYGB, SADI, and gastric banding using varied methods of intraperitoneal instillation [12–16, 21]. Most studies demonstrate a significant improvement in pain immediately post-operatively when utilizing the intraperitoneal instillation method with which the current study’s results are consistent. In a study using intraperitoneal ropivacaine by Ruiz-Tovar et al. [13], reduced post-operative pain scores, lower morphine consumption, earlier time to mobilization, and shorter hospital stay were observed in patients undergoing SG and RYGB. Intraperitoneal instillation has also been combined with surgical site injection of bupivacaine with significantly prolonged time to first post-operative analgesia request in the treatment group [12]. Analgesic effects of intraperitoneal bupivacaine have even been extended to 2 h post-operatively when an increased dose is used and the patient is kept in the Trendelenburg position for 5 min post instillation [14]. A study by Schipper et al. [21] is one of very few which has found no significant analgesic effects of intraperitoneal bupivacaine when observing pain score and opioid use.

Although the analgesic effects of intraperitoneal local anesthetic are observed in the majority of studies, methodology was variable. There are variances in the primary outcomes and methods used to assess these, as well as the type of local anesthetic used. Most studies have used bupivacaine [12, 14–19, 21] with very few using ropivacaine as an option [13]. Ropivacaine was the local anesthetic of choice in this study due to its decreased cardiotoxicity and central nervous system toxicity when compared with bupivacaine [20]. Similar to Ruiz-Tovar et al. [13] who also used ropivacaine as their local anesthetic, the current study demonstrated significantly reduced post-operative pain scores.

We chose to include a range of primary and revision procedures in our study, which increases the generalizability of our results. In addition, all procedures were carried out by the same surgeon and anesthetist minimizing variation in procedural technique and anesthetic protocol. A standardized approach was maintained when administering the local anesthetic solution by calculating dosage at 0.5 mL/kg according to weight on the day of surgery. This ensured that patients were receiving safe and proportionate amounts of analgesia which is particularly important when considering substantial weight variations in patients with obesity. Our study uses a simple method of instillation utilizing a simple spraying cannula attached to a 20 mL syringe. This is readily available, adds little time to the operation, and avoids using expensive and complex devices to aerosolize or nebulize local anesthetic.

### Limitations

Insufflation rate is an important consideration as Ozdemir et al. [22] argue that a lower flow rate and pressure can reduce pain scores post-operatively. The current study used a flow rate of 10 L/min with a pressure of 14 mmHg which is towards the higher end of recommended pressures [22]; however, low insufflation pressure and rate decrease the view for the surgeon thus potentially prolonging the procedure.

Preoperative analgesia requirements due to chronic pain are an important consideration. Interestingly, in the present analysis, a diagnosis of chronic pain and associated preoperative analgesia use was not associated with post-operative pain scores on uni- or multivariable analysis.

Future studies could address whether both low insufflation rates and pressures, as well as a brief period of Trendelenburg positioning post instillation, enhance the analgesic effect that we have confirmed.

## Conclusion

This study demonstrated that the administration of ropivacaine intraperitoneally during laparoscopic bariatric surgery is simple to perform, safe, and reduces post-operative pain and severe pain episodes in the recovery room. However, there was no difference in total opioid or antiemetic use nor LOS.

## Supplementary Information

Below is the link to the electronic supplementary material.

Supplementary file1 (DOCX 16 KB)

Supplementary file2 (DOCX 15 KB)

Supplementary file3 (DOCX 15 KB)
