# Supplementary material for: Intraperitoneal Instillation of Local Anesthetic (IPILA) in Bariatric Surgery and the Effect on Post-operative Pain Scores: a Randomized Control Trial
Source: Obes Surg. 2022 May 4;32(7):2349–56. doi: 10.1007/s11695-022-06086-w (PMC9276555; doi:10.1007/s11695-022-06086-w)
Supplement: Supplementary file 1 — Supplementary file1 (DOCX 16 KB) [file 11695_2022_6086_MOESM1_ESM.docx]

| **Supplementary Table 1. Anaesthetic baseline data** | | |  |  |
| --- | --- | --- | --- | --- |
|  | **Total (N=104)** | **IPILA (N=50)** | **Placebo (N=54)** | **p-value** |
| **TIVA** |  |  |  | 0.695 |
| Yes | 104 (100.0%) | 50 (100.0%) | 54 (100.0%) |  |
| **Inhaled vapour anaesthetic** |  |  |  | 0.695 |
| Yes | 104 (100.0%) | 50 (100.0%) | 54 (100.0%) |  |
| **Muscle relaxant** |  |  |  | 0.334 |
| No | 1 (1.0%) | 0 (0.0%) | 1 (1.9%) |  |
| Yes | 103 (99.0%) | 50 (100.0%) | 53 (98.1%) |  |
| **Opiod anaesthetic** |  |  |  | 0.821 |
| No | 78 (75.0%) | 38 (76.0%) | 40 (74.1%) |  |
| Yes | 26 (25.0%) | 12 (24.0%) | 14 (25.9%) |  |
| **Opiod quantification** |  |  |  | 0.154 |
| fentanyl (non-documented dose) | 1 (4.0%) | 1 (9.1%) | 0 (0.0%) |  |
| fentanyl 150mg | 21 (84.0%) | 10 (90.9%) | 11 (78.6%) |  |
| fentanyl 175mg | 3 (12.0%) | 0 (0.0%) | 3 (21.4%) |  |
| **Regional block** |  |  |  | 0.695 |
| No | 104 (100.0%) | 50 (100.0%) | 54 (100.0%) |  |
| **Nitrous oxide use** |  |  |  | 0.695 |
| No | 104 (100.0%) | 50 (100.0%) | 54 (100.0%) |  |
| **Adjuncts (non-specified)** |  |  |  | 0.695 |
| Yes | 104 (100.0%) | 50 (100.0%) | 54 (100.0%) |  |
| **Tramadol use** |  |  |  | 0.695 |
| Yes | 104 (100.0%) | 50 (100.0%) | 54 (100.0%) |  |
| **Parecoxib** |  |  |  | 0.695 |
| Yes | 104 (100.0%) | 50 (100.0%) | 54 (100.0%) |  |
| **Ketamine** |  |  |  | 0.695 |
| Yes | 104 (100.0%) | 50 (100.0%) | 54 (100.0%) |  |
| **Caldolor** |  |  |  | 0.695 |
| No | 104 (100.0%) | 50 (100.0%) | 54 (100.0%) |  |
| **Antiemetic use** |  |  |  | 0.937 |
| No | 4 (3.8%) | 2 (4.0%) | 2 (3.7%) |  |
| Yes | 100 (96.2%) | 48 (96.0%) | 52 (96.3%) |  |
