# Supplementary material for: Intraperitoneal Instillation of Local Anesthetic (IPILA) in Bariatric Surgery and the Effect on Post-operative Pain Scores: a Randomized Control Trial
Source: Obes Surg. 2022 May 4;32(7):2349–56. doi: 10.1007/s11695-022-06086-w (PMC9276555; doi:10.1007/s11695-022-06086-w)
Supplement: Supplementary file 2 — Supplementary file2 (DOCX 15 KB) [file 11695_2022_6086_MOESM2_ESM.docx]

| Supplementary Table 2. Uni- and multivariable analysis of factors influencing mean changes in VAS scores at rest | | | | | |
| --- | --- | --- | --- | --- | --- |
|  |  | **Univariable coefficient (95%CI)** | **p-value** | **Multivariable coefficient (95%CI)** | **p-value** |
| IPILA | No |  | - |  | - |
|  | Yes | -1.01 (-2.14–0.13) | 0.082 | -0.88 (-2.07–0.32) | 0.148 |
| Surgery type | LSG |  | - |  | - |
|  | OAGB | 1.56 (-0.20–3.32) | 0.082 | 1.91 (0.14–3.68) | 0.035 |
|  | RYGB | 0.19 (-1.52–1.89) | 0.829 | 0.75 (-1.05–2.54) | 0.41 |
|  | SADI | 0.83 (-2.17–3.83) | 0.585 | 1.04 (-1.98–4.06) | 0.494 |
| Concomittant hiatus hernia repair | No |  | - |  | - |
|  | Yes | -1.35 (-2.64–-0.06) | 0.041 | -1.07 (-2.49–0.36) | 0.14 |
| Age |  | -0.03 (-0.08–0.01) | 0.162 | -0.02 (-0.08–0.03) | 0.383 |
| BMI |  | 0.05 (-0.03–0.14) | 0.228 | 0.06 (-0.03–0.16) | 0.209 |
| Preoperative chronic pain | No |  | - |  | - |
|  | Yes | -0.22 (-2.09–1.65) | 0.817 | 0.32 (-1.72–2.36) | 0.756 |
