# Supplementary material for: Intraperitoneal Instillation of Local Anesthetic (IPILA) in Bariatric Surgery and the Effect on Post-operative Pain Scores: a Randomized Control Trial
Source: Obes Surg. 2022 May 4;32(7):2349–56. doi: 10.1007/s11695-022-06086-w (PMC9276555; doi:10.1007/s11695-022-06086-w)
Supplement: Supplementary file 3 — Supplementary file3 (DOCX 15 KB) [file 11695_2022_6086_MOESM3_ESM.docx]

| **Supplementary Table 3. Uni- and multivariable analysis of factors influencing mean changes in VAS scores on movement** | | | | | |
| --- | --- | --- | --- | --- | --- |
|  |  | **Univariable coefficient (95%CI)** | **p-value** | **Multivariable coefficient (95%CI)** | **p-value** |
| **IPILA** | No |  | - |  | - |
|  | Yes | -1.21 (-2.44 to 0.03) | 0.055 | -1.05 (-2.34 to 0.24) | 0.109 |
| **Surgery type** | LSG |  | - |  | - |
|  | OAGB | 1.57 (-0.34 to 3.48) | 0.107 | 2.03 (0.12 to 3.94) | 0.038 |
|  | RYGB | 0.46 (-1.40 to 2.31) | 0.626 | 1.11 (-0.83 to 3.05) | 0.259 |
|  | SADI | 1.28 (-1.98 to 4.54) | 0.438 | 1.35 (-1.91 to 4.61) | 0.414 |
| **Concomittant hiatus hernia repair** | No |  | - |  | - |
|  | Yes | -1.07 (-2.49 to 0.35) | 0.139 | -0.63 (-2.16 to 0.91) | 0.421 |
| **Age** | [18,67] | -0.05 (-0.10 to 0.00) | 0.062 | -0.04 (-0.10 to 0.01) | 0.143 |
| **BMI** | [29,61.1] | 0.06 (-0.04 to 0.16) | 0.214 | 0.07 (-0.03 to 0.17) | 0.19 |
| **Preoperative chronic pain** | No |  | - |  | - |
|  | Yes | -0.42 (-2.45 to 1.61) | 0.685 | 0.30 (-1.90 to 2.50) | 0.787 |
